# Perspectives on Preimplantation Genetic Testing for Monogenic Disorders Among Japanese Patients With Hereditary Breast Cancer Undergoing Fertility Preservation: Insights From the First Japanese Survey

**DOI:** 10.1002/rmb2.12678

**Published:** 2025-09-17

**Authors:** Haruhisa Konishi, Yoshiharu Nakaoka, Anmae Michiko, Sho Fujiwara, Rie Kitayama, Daisuke Kadogami, Naoharu Morimoto, Kanako Katsu, Satoko Fujioka, Tomoko Inoue, Aisaku Fukuda, Hiroki Kurahashi, Yoshiharu Morimoto

**Affiliations:** ^1^ IVF Namba Clinic Osaka Japan; ^2^ IVF Osaka Clinic Osaka Japan; ^3^ HORAC GRANDFRONT IVF Osaka Clinic, Grand Front Osaka Tower B Osaka Japan; ^4^ Division of Molecular Genetics Center for Medical Science Fujita Health University Toyoake Aichi Japan

**Keywords:** adolescent and young adult populations, *BRCA1/2*, fertility preservation, preimplantation genetic testing, reproductive‐decision‐making

## Abstract

**Purpose:**

Preimplantation genetic testing for monogenic disorders (PGT‐M) offers *BRCA* variant carriers the option of preventing hereditary cancer transmission. We investigated the awareness and attitudes toward PGT‐M among patients with breast cancer who underwent fertility preservation.

**Methods:**

A questionnaire‐based survey was administered to 264 patients with breast cancer who were eligible for oocyte or embryo cryopreservation at in vitro fertilization clinics between October 2024 and March 2025. A total of 161 valid responses were analyzed. The survey assessed *BRCA* testing status, PGT‐M awareness, willingness to undergo PGT‐M, and opinions on future availability.

**Results:**

The uptake rate of *BRCA1/2* testing was 53.4%; 14% of the respondents were variant carriers. Only 16.8% had prior awareness of PGT‐M, and 47.8% expressed a willingness to use PGT‐M if available. Among *BRCA* ‐variant carriers, 3.3% reported that they would consider PGT‐M, and 75% believed it should be made available upon request. Overall, 68.3% supported information sharing between oncology and fertility providers.

**Conclusion:**

These findings highlight the importance of expanding reproductive options and patient awareness of PGT‐M in the care of patients with hereditary cancer. Discussions should focus on how best to provide accurate information and enable informed reproductive choices for those at genetic risk.

## 1 Introduction

Advances in assisted reproductive technology (ART), including preimplantation genetic testing for monogenic disorders (PGT‐M), have made it possible to avoid genetic transmission of disease‐causing genetic variants [1]. In recent years, addressing reproductive concerns among adolescent and young adult (AYA) patients with cancer has garnered significant interest. Among these patients, individuals with hereditary breast and ovarian cancer syndrome (HBOC), caused by pathogenic variants in *BRCA1* or *BRCA2*, face not only elevated cancer risks but also difficult reproductive decisions regarding the potential transmission of these variants to their offspring [2]. In 2003, acknowledging the 50% inheritance risk and its implications for reproductive decision‐making, the Ethics Task Force of the European Society of Human Reproduction and Embryology (ESHRE) endorsed the use of preimplantation genetic testing (PGT) for late‐onset or incompletely penetrant conditions, including HBOC related to *BRCA1/2* pathogenic variants [3, 4]. The ethical acceptability of PGT‐M for adult‐onset diseases such as HBOC has gained wide global recognition. According to the ESHRE, HBOC was the second most common indication for PGT‐M in 2018 [5]. In contrast, the current regulations in Japan limit the use of PGT‐M to conditions that severely impair daily functioning or survival before adulthood. To date, no formal application has been submitted for the use of PGT‐M in hereditary cancer syndromes, including *BRCA*‐related conditions [6]. Moreover, no patient‐centered surveys exploring the awareness of or attitudes toward PGT‐M have been conducted in Japan. Consequently, it remains unclear whether patients are aware of this option, lack optimism, have abandoned the possibility, or have considered accessing PGT‐M overseas.

Fertility preservation therapy is a crucial consideration for patients undergoing cancer treatment because of its possible impact on reproductive potential. This study aimed to investigate the uptake of *BRCA1/2* genetic testing and evaluate the awareness of and attitudes toward PGT‐M among patients with breast cancer who have undergone fertility preservation. The goal was to capture the perspectives of patients and help oncologists and reproductive specialists better understand their reproductive autonomy and the evolving role of genetics in reproductive decision‐making. Ultimately, we hope that our findings will support the development of a clinical framework for implementing PGT‐M. To our knowledge, this is the first patient‐centered survey in Japan focusing on PGT‐M awareness in the context of hereditary cancer syndromes.

## 2 Materials and Methods

A questionnaire‐based survey was conducted among 264 patients with breast cancer who underwent fertility preservation and were eligible for oocyte or embryo cryopreservation between January 2014 and December 2023 at the IVF Namba Clinic and its affiliated facilities (HORAC Grand Front Osaka Clinic, IVF Osaka Clinic). This study was approved by the Ethics Committee of the IVF JAPAN group (Approval Number: 2024‐15). The survey was open to responses between October 2024 and March 2025. Participants received anonymized, self‐administered questionnaires distributed via postal mail or Google Forms. A total of 161 valid responses were collected, yielding a response rate of 61.0% (55 via mail and 106 via online submission).

**FIGURE 1:**
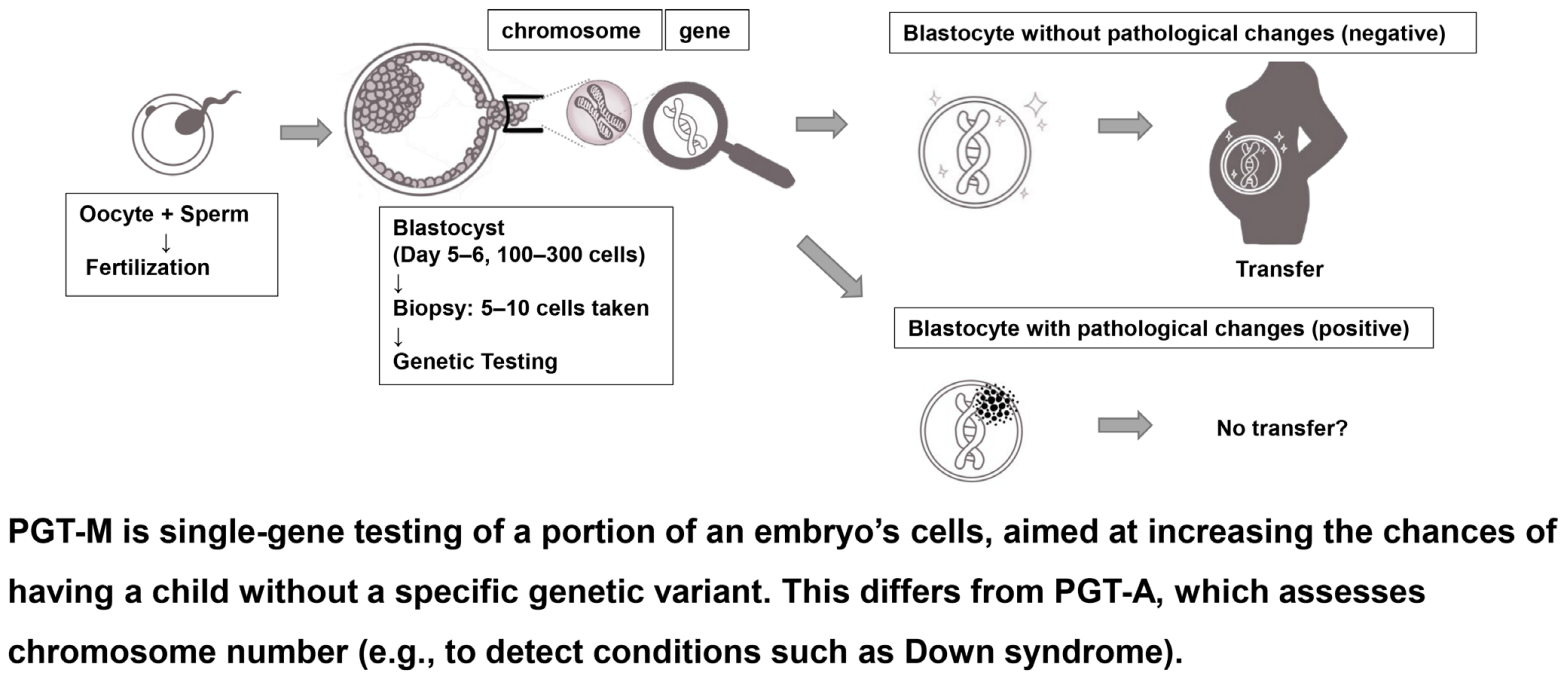
Schematic explanation of PGT‐M shown to the participants. A schematic explanation of the PGT‐M process, including embryo biopsy and genetic testing. This figure was shown to all participants prior to questions about their awareness and acceptability of PGT‐M. It also clarified the distinction between PGT‐M (single‐gene testing) and PGT‐A (chromosomal testing).

The survey assessed several key aspects of genetic testing and reproductive decision making. The four main outcomes included: (i) whether *BRCA1/2* genetic testing had been performed and the results; (ii) awareness of PGT‐M for hereditary cancer syndrome, after viewing an explanatory figure (Figure 1); (iii) willingness to undergo PGT‐M if available; and (iv) opinions regarding whether PGT‐M for hereditary cancer syndrome should be available upon patient request in the future.

Demographic and clinical information was also collected, including age, parity (presence or absence of children), marital/partner status, duration of cancer treatment, current treatment status, family history of cancer, and pregnancy expectations. At the end of the survey, participants were asked whether they felt it was necessary to share genetic test results and other medical information with fertility preservation and cancer treatment providers.

Descriptive statistics were used to summarize participant characteristics and survey responses. Chi‐squared tests were used to compare responses between *BRCA* variant carriers and other participants. Statistical analyses were performed using Microsoft Excel (Microsoft Corporation, Redmond, WA, USA), and a *p*‐value < 0.05 was considered statistically significant.

## 3 Results

### 3.1 Participant Characteristics

**FIGURE 2:**
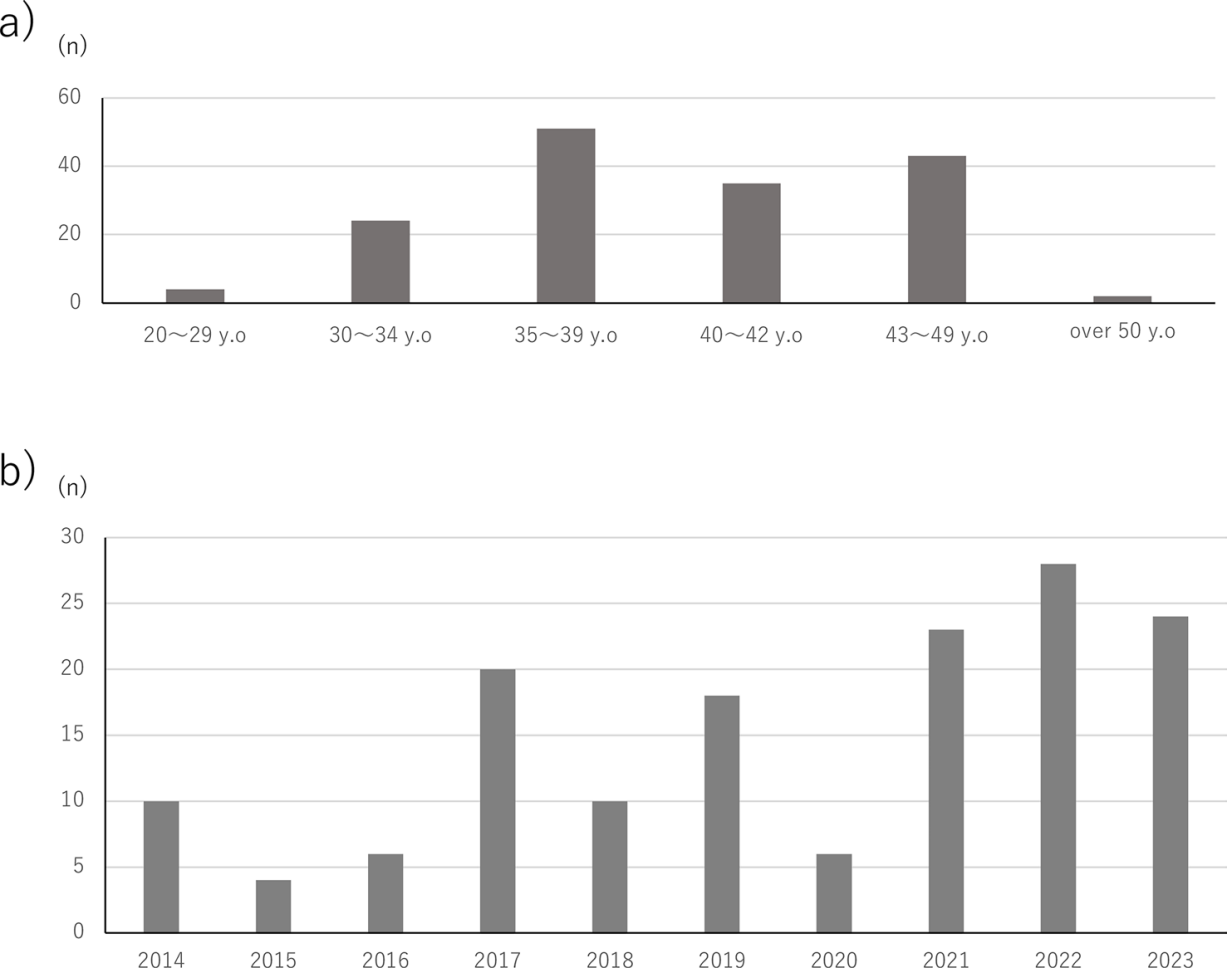
Participant background. (a) Age distribution of participants at the time of survey. The median age was 40.2 years, based on grouped age data. Most participants were in their late 30s to early 40s, which corresponds to the typical reproductive age range for fertility preservation candidates in Japan. (b) Distribution of breast cancer treatment year among participants. The median treatment year was 2021, and the majority (59.1%) received cancer treatment in 2020 or later. This timing reflects recent clinical developments, including expanded insurance coverage for *BRCA* testing and increased awareness of fertility preservation options.

The distribution of participant age and duration of cancer treatment is shown in Table 1 and Figure 2a,b. While 14.9% of the respondents reported no intention of future childbearing, over 80% indicated a desire to continue oocyte or embryo cryopreservation or to pursue reproductive treatment for future pregnancies. Regarding cancer treatment status, 60.2% were undergoing active treatment (including maintenance hormonal therapy), whereas 40% were being monitored.

**TABLE 1:** Participant characteristics.

	Number (%)
Parity	
No children	118 (73.3)
One child	35 (21.7)
Two or more children	6 (3.7)
Prefer not to answer	2 (1.2)
Marital/Partner Status	
Married	87 (54)
Unmarried (with partner)	14 (8.7)
Unmarried (without partner)	60 (37.3)
Current cancer treatment status	
Completed treatment (under follow‐up)	64 (39.8)
Under treatment (including hormonal therapy)	97 (60.2)
Family history of cancer	
First‐degree relatives with the same type of cancer	31 (19.3)
First‐degree relatives with a different type of cancer	38 (23.6)
No family history of cancer	90 (55.9)
Prefer not to answer	2
Future pregnancy plans	
Not considering future pregnancy	24 (14.9)
Currently pregnant (including recently postpartum)	7 (4.3)
Planning natural conception or new fertility treatment	8 (5)
Planning pregnancy using frozen oocytes or embryos	34 (21.1)
Planning to extend storage of frozen oocytes (no current plan for use)	71 (44.1)
Planning to extend storage of frozen embryos (no current plan for use)	17 (10.6)
Have you heard of or are you aware of BRCA testing?	
Yes	115 (71.4)
No	46 (28.6)
Have you undergone BRCA genetic testing?	
Yes	86 (53.4)
Plan to undergo	4 (2.5)
Do not plan to undergo	25 (15.5)
Not sure	46 (28.6)
Result of BRCA testing (*n* = 85)	
BRCA1 positive	5 (5.8)
BRCA2 positive	4 (4.7)
Positive (unspecified type)	3 (3.5)
Negative	71 (82.6)
Prefer not to answer/Blank	3 (3.5)

Regarding family history, 19.3% had a first‐degree relative with breast cancer, 23.6% had a relative with another type of cancer, and 55.9% had no known family history.

### 3.2 BRCA Testing Status

The overall uptake rate of *BRCA1/2* genetic testing was 53.4%. Among those who had not undergone testing, 28.6% indicated that they had never heard of the *BRCA* genes. Among those tested, 14% carried pathogenic variants, 5.8% in *BRCA1* and 4.7% in *BRCA2*.

### 3.3 Awareness and Consideration of PGT‐M

After participants were presented with an explanatory figure (Figure 1), only 16.8% reported prior awareness of PGT‐M. Among those with pathogenic *BRCA* variant carriers, two (16.8%) were aware of PGT‐M. All 27 participants who reported prior awareness of PGT‐M were aged 35 years or older. The proportion of awareness increased with age: 15.7% in those aged 35–39, 16.7% in those aged 40–42, 25.0% in those aged 43–49, and 100% in those aged 50 or older. No apparent association was found between PGT‐M awareness and *BRCA* status, year of cancer diagnosis, or family history of cancer.

When asked about their willingness to undergo PGT‐M if it were available, 47.8% of all respondents expressed interest (Table 2). Among the *BRCA* variant carriers, 33.3% expressed willingness to use PGT‐M. Awareness and willingness were comparable regardless of *BRCA* status.

**TABLE 2:** Survey responses among all participants and *BRCA* variant carriers.

	All (*n* = 161) (%)	BRCA variant (*n* = 12) (%)
Had you heard of or were you aware of PGT‐M before this survey?		
Yes	27 (16.8)	2 (16.8)
No	134 (83.2)	10 (83.3)
If PGT‐M were available, would you want to undergo it?		
Yes	77 (47.8)	4 (33.3)
No	6 (3.7)	0
Not sure	78 (48.4)	8 (66.7)
If PGT‐M were performed and the result indicated a pathogenic variant, would you choose to transfer the embryo?		
Yes*	6 (3.7)	2 (16.7)
No	55 (34.2)	5 (41.7)
Not sure	92 (57.1)	5 (41.7)
Other	8 (5)	0
Opinion on future availability of PGT‐M for hereditary cancers		
Should be available upon request*	70 (43.5)	9 (75)
No need to make it available	2 (1.2)	0
Not sure	86 (53.4)	3 (25)
Other	3 (1.9)	0
Should BRCA test results be shared between fertility preservation and cancer treatment facilities?		
Yes	110 (68.3)	6 (50)
No	9 (5.6)	1 (8.3)
Not sure	42 (26.1)	5 (41.7)

*Note:* “Yes” responses were compared between *BRCA* variant carriers (*n* = 12) and the remaining participants (*n* = 149), excluding the variant group from the total. Items marked with * showed statistically significant differences between the two groups: (*p* < 0.05). (i) Willingness to transfer an embryo with a known pathogenic variant. (ii) Support for the future availability of PGT‐M upon request. No significant differences were found for other items. “No” and “Not sure” responses were not included in the statistical analysis.

When asked whether they would consider transferring a *BRCA*‐positive embryo, 3.7% answered “yes,” 34.2% said “no,” and 57.1% were “unsure.”

### 3.4 Acceptability of PGT‐M

**FIGURE 3:**
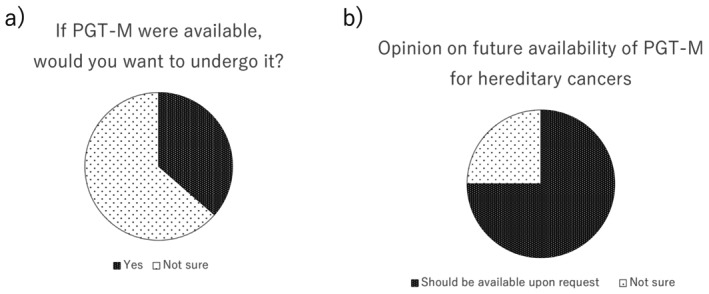
Key attitudes toward PGT‐M among *BRCA* variant carriers (*n* = 12). (a) Personal willingness to undergo PGT‐M if it were available. (b) Support for future availability of PGT‐M upon patient request. Among these *BRCA* variant carriers, only 33.3% expressed a personal willingness to use PGT‐M, while 75.0% supported making it available in principle. This marked discrepancy reflects a recurring theme in reproductive ethics: Even individuals who would not choose PGT‐M for themselves may strongly value the right of others to access it.

Participants were asked the following question: “In Japan, the current criteria for PGT‐M generally limit its use to conditions that severely affect daily life or survival before adulthood. As a result, hereditary cancers, such as those associated with *BRCA* variants, which typically manifest in adulthood, have not yet been included in PGT‐M. What are your thoughts about this policy?” Overall, 43.5% responded that PGT‐M should be made available upon patient request, 53.4% were unsure, and only 1.2% responded that there was no need for future implementation (Table 2). Notably, among the *BRCA* variant carriers, 75% expressed support for the future availability of PGT‐M upon request, indicating a higher level of acceptance among those at direct genetic risk (Table 2, Figure 3).

### 3.5 Opinions on Medical Information Sharing

Regarding the integration of medical care, participants were asked whether they thought it was necessary to share genetic testing results and other medical information with fertility preservation and cancer treatment providers. Of the respondents, 68.3% answered “necessary,” 5.6% said “unnecessary,” and 26.1% were “unsure.”

For statistical analysis, *BRCA* variant carriers (*n* = 12) were compared with the remaining participants (*n* = 149), excluding the carriers from the total cohort.

Statistically significant differences were observed for two items:

1. “If PGT‐M indicated a pathogenic variant, would you transfer the embryo?”, and
2. “Should PGT‐M be made available upon patient request?”

No significant differences were found on other survey items. Responses marked as “No” or “Not sure” were excluded from the statistical analysis.

## 4 Discussion

In the present study, the uptake rate of *BRCA1/2* genetic testing among patients with breast cancer who had undergone fertility preservation was 53.4%. This rate is expected to increase as insurance coverage for *BRCA* testing was gradually expanded in Japan between 2018 and 2020. Moreover, the increasing use of multigene panel testing has contributed to the identification of pathogenic variants associated with hereditary cancer syndromes. The proportion of *BRCA* variant carriers in this study was 14%, which is consistent with previously reported rates of approximately 15% in adolescent and young adult populations [7]. Our findings suggest that the presence of a pathogenic *BRCA* variant does not discourage patients from pursuing fertility preservation. Prior studies have shown that *BRCA* status can influence reproductive decision‐making [1, 8]. Among women who had not completed childbearing at the time of *BRCA* test disclosure, 17% chose not to have children because of concerns about genetic transmission, 43% decided to have children earlier, and 30%–40% considered ART or oocyte cryopreservation [2]. As genetic testing becomes more widespread and cancer survivorship improves, reproductive counseling is likely to become increasingly important for *BRCA* variant carriers. These findings underscore the requirement for an integrated system that supports both reproductive medicine and genetic counseling for individuals with hereditary cancer syndromes.

In our study, only 16.8% of participants were aware of PGT‐M. Previous reports have emphasized the importance of providing genetic counseling, which includes information on reproductive options, such as PGT‐M and prenatal diagnosis [9]. Many patients have expressed a desire to receive standardized information about *BRCA*‐related reproductive options during oncogenic counseling, regardless of whether they ultimately pursue those options [10]. However, recognition of the psychological burden associated with PGT‐M is also important, as patients may feel pressured to make critical decisions quickly, experience emotional conflict involving their partners and family, or feel guilt over the potential transmission of a pathogenic variant. In addition, personal health concerns and the perceived treatment risk may further complicate decision‐making [10]. This emotional burden is often intensified when *BRCA* disclosure and fertility‐related information are provided simultaneously [11, 12, 13]. In our study, 16.7% of *BRCA* variant carriers responded that they would choose to transfer an embryo even if it tested positive for a pathogenic variant, compared to only 3.4% of the remaining participants. This contrast suggests that being a *BRCA* carrier may shape one's reproductive attitudes and risk perception in unique ways. Standardized information alone may be insufficient; flexible and responsive support that considers each patient's personal context and values may be equally important. Furthermore, fragmented care between oncology and reproductive services can also contribute to patient confusion and stress. In our study, 68.3% of participants stated that sharing genetic and medical information across institutions was necessary. These findings highlight the importance of inter‐institutional collaboration in supporting patient autonomy and promoting the development of effective reproductive counseling systems.

A notable finding of this study was the discrepancy between personal willingness to undergo PGT‐M and the broader acceptance of its availability. While 33.3% of participants with *BRCA* positivity reported that they would consider undergoing PGT‐M, 75% believed that it should be made available upon request (Figure 3a,b). This pattern is consistent with that reported in previous studies published over the past decade [14]. Many reports have shown that, although a large proportion of *BRCA* variant carriers support PGT as a reproductive option in principle, fewer are willing to pursue it. The relatively low level of willingness to undergo PGT‐M may be influenced by several factors, including limited awareness among both patients and healthcare providers, lack of availability in the domestic healthcare system, concerns about cost, treatment burden, success rates, and anxiety regarding pregnancy after breast cancer [15]. This gap between personal considerations and policy‐level acceptability may reflect a more general human tendency to value reproductive autonomy, even if individuals do not exercise all available options. Regardless of whether patients ultimately choose to use PGT‐M, many expressed a desire for the freedom to make informed choices.

This study focused on a specific population of patients with breast cancer who underwent fertility preservation, making them highly relevant to the discussion of PGT‐M. Among the 14% who carried pathogenic *BRCA* variants, 75% expressed support for making PGT‐M available in the future, whereas in the overall cohort, only 43.5% supported it and 53.5% responded “not sure”. This contrast suggests that individuals with *BRCA* pathogenic variants are more likely to engage with the issue directly, perceiving the risk of hereditary cancer transmission both personally and concretely.

PGT‐M has been widely explored for its clinical utility and cost‐effectiveness in the treatment of hereditary cancer syndromes [13]. Although several studies have reported reduced ovarian reserve markers, such as lower AMH levels and decreased oocyte maturation rates, in *BRCA* variant carriers, the total number of oocytes retrieved did not appear to differ significantly [14, 15, 16, 17]. These findings support the feasibility of preserving fertility in patients who are *BRCA* variant carriers. Moreover, the selection of *BRCA*‐negative embryos using PGT‐M may eliminate the need for lifelong surveillance of potential carriers and reduce long‐term healthcare costs. Taken together, these observations support the clinical viability and technical reliability of ART and PGT‐M for *BRCA*‐associated hereditary cancer syndromes.

This study has several limitations. First, the participants were limited to patients with breast cancer who had undergone fertility preservation, which may reflect a specific subset of individuals with heightened reproductive awareness or interest. Additionally, the survey was conducted at least one year after the start of cancer treatment, which may have influenced respondents' attitudes and decision‐making retrospectively. Second, while this study focused on *BRCA*‐related HBOC, other hereditary cancer syndromes such as Li‐Fraumeni syndrome, von Hippel–Lindau disease, or familial adenomatous polyposis are often diagnosed at much younger ages. For patients with these conditions, perspectives on reproduction and preimplantation genetic testing may differ significantly. Therefore, the findings of this study may not be directly applicable to those populations. Third, the sample size was relatively limited and derived from patients attending a single IVF clinic group, which may affect the generalizability of the findings to broader populations. Further research involving a broader range of hereditary cancer syndromes and treatment timelines is warranted to better capture diverse patient needs and preferences regarding PGT‐M.

In conclusion, as genetic technologies continue to advance and more individuals are identified as carriers of hereditary cancer variants, even before disease onset, the demand for both fertility preservation and PGT‐M is likely to increase. Based on the patient‐centered survey results, the first undertaken in Japan, future research and policy‐making should focus on the development of a clinical and ethical framework that addresses the reproductive needs of individuals with hereditary cancer syndromes while ensuring informed decision making.

## Ethics Statement

All procedures followed were in accordance with the ethical standards of the Ethics Committee of the IVF JAPAN group (approval number: 2024‐15) and were conducted in accordance with the Declaration of Helsinki of 1964 and its later amendments.

## Consent

Written informed consent was not required, as participation in the survey was voluntary and responses were collected anonymously. The questionnaire included an introductory statement indicating that submission of the completed form would be regarded as consent to participate.

## Conflicts of Interest

The authors declare no conflicts of interest. Yoshiharu Morimoto is an Editorial Board member of Reproductive Medicine and Biology and a co‐author of this article. To minimize bias, he was excluded from all editorial decision‐making related to the acceptance of this article for publication.

## Data Availability

The data that support the findings of this study are available from the corresponding author upon reasonable request. We plan to deposit the dataset in a public repository upon acceptance.
